# Behavioral intentions of rural farmers to recycle human excreta in agriculture

**DOI:** 10.1038/s41598-022-09917-z

**Published:** 2022-04-07

**Authors:** Simon Gwara, Edilegnaw Wale, Alfred Odindo

**Affiliations:** 1 Discipline of Agricultural Economics, School of Agricultural, Earth and Environmental Sciences, University of KwaZulu-Natal, Pietermaritzburg, 3201 South Africa; 2 Department of Agricultural Economics, Faculty of Natural and Agricultural Sciences, University of the Free State, Bloemfontein, 9300 South Africa; 3 Discipline of Crop Science, School of Agricultural, Earth and Environmental Sciences, University of KwaZulu-Natal, Pietermaritzburg, 3201 South Africa

**Keywords:** Environmental social sciences, Environmental economics, Psychology and behaviour, Socioeconomic scenarios, Sustainability

## Abstract

Considerable progress has been made in developing human excreta recovery pathways and processes for maximum nutrient recovery and contaminant elimination. The demand segment has often been ignored as an area for future research, especially during the technology development. The findings from the few published articles on social acceptance show missing and inconclusive influence of demographic, sociological, and economic farmer-characteristics. This study endeavours to close this gap by using the social psychological theories, technology adoption theories and the new ecological paradigm to investigate the factors that influence the behavioral intentions of rural farmers to recycle human excreta in agriculture. Study findings show that social acceptance was driven by awareness, religiosity, income, source of income, and environmental dispositions. Perceived behavioral control represents a potential barrier to human excreta reuse. The study recommends the demographic, cultural, sociological, and economic mainstreaming of dissemination strategies of circular bioeconomy approaches within the context of agricultural innovation systems.

## Introduction

The global demand for food is soaring due to rapid population growth, urbanization, and international trade^1,2^. The decline in soil fertility in sub-Saharan Africa is continues to threaten household-level food security^3,4^. The mining of soil nutrients via food transportation from farms is worsened by rapid population, urbanization, economic development, rising incomes, and nutrition transition^5,6^. Plant nutrients are involved in anabolic processes that produce organic compounds during photosynthesis and are not absorbed by a healthy human body, but excreted as, for instance, faeces and urine. The mining of nutrients from the agricultural soils via food creates nutrient sinks in urban environments^6^. Annual nutrient mining rates in Africa ranges from 9 to 88 kg NPK/ha^7,8^. About 60–70% of the nutrients mined from the soil as food, and through soil erosion, leaching and as human excreta goes to the environment and closing the nutrient loop would restore the ecological balance and soil health^9^. Nutrient mining may also result in long-term productivity failure^6^ and may pose negative health impacts related to micronutrient deficiency in developing countries^8^. The use of chemical fertilizers is often considered the only viable option to supply plant nutrients^3,10^. On the other hand, the average annual fertilizer application rates (at lowest 8 kg/ha) in sub-Saharan Africa are far below the nutrient depletion rates or the plant requirements for crop production^11^.

The sub-Saharan Africa uses annual chemical fertilizer application rates of as low as 8 kg/ha/year, far below the nutrient depletion rates or the plant requirements for crop production^11^. The mean fertilizer application rate in Sub-Saharan Africa is about 8 kg/ha/year and far below the 141 kg/ha/year in South Asia, 154 kg/ha/year in the Europe, 175 kg/ha/year in South America, and 302 kg/ha/year in East Asia^12^. The excessive use of chemical fertilizers poses negative environmental impacts on aquatic systems and soil health^13–15^, as well as to human health^16,17^. Globally, sustainable food production under the high-input agricultural intensification systems, which mainly depends on intensive use of synthetic fertilizers is considered impossible without causing significant negative environmental impacts^18,19^. Ecological intensification, therefore, has a great potential to achieve sustainable food production without using external inputs^20^. The use of sustainable agricultural systems to restore soil health augments agricultural productivity, reduces greenhouse emissions, and build the soil's resilience to shocks^21^.

In rural South Africa, farmers face challenges of sanitation where approximately three million ventilated improved pit latrines constructed by the government in response to the millennium development goals for universal sanitation in the last 15 years are currently filled-up. The majority of the local authorities neither have a policy, plan, nor budget for Faecal Sludge Management (FSM) for instance, emptying, transportation and disposal of faecal sludge^22^. The common practice is the building of inferior makeshift pits (rather than emptying and reuse) which expose farmers to known health risks. In instances where emptying is possible, there are considerable environmental impacts and space constraints associated with the dumping of waste in hazardous landfills. Deep row entrenchment often considered a simpler and immediate solution to faecal sludge disposal from on-site sanitation systems^22^. More research is needed to understand the mineralization (ammonification and nitrification) processes in deep row entrenchment. For instance, examining the extent to which the conditions at depth would allow for nitrification of ammonium to inorganic compounds for absorption. Hazardous landfills and deep trenches should be far from residents to reduce air pollution, but close enough to the waste source to reduce transportation costs^23^, with the trade-off being met with high landfill costs and stringent environmental regulations.

Technologies designed to safely recover agricultural nutrients from human faeces include biochar pyrolysis^24,25^, black soldier fly^26,27^, Latrine biosolids Dehydration and Pasteurization (LaDePa)^28^, and co-composting and vermicomposting^14,29^. Acid extraction of phosphorus from incineration ash (RecoPhos®), phosphorus crystallization of digester supernatant (AirPrex® and Ostara®) and composting have the highest technology readiness levels^30,31^. Co-composting is, however, preferred as a low-cost technology^32^, and due to its link with the “circular sanitation economy in agriculture” school of thought^33^. The composting thermophilic biological degradation process can inactivate helminth eggs to the World Health Organisation’s recommended levels for safe agricultural use^34,35^. Considerable studies have demonstrated the benefits of co-compost application on land in terms of improved soil water-holding capacity, nutrient retention, and soil structure^36–39^. Compost application complements and supplements the use of chemicals fertilizers by augmenting their agronomic efficiency^4,10,40^.

The co-composting value chain, however, continues to face potential barriers related to lack of wide-scale acceptance of the end product by customers^41^. A synthesis of nutrient management systems attributes the low demand for fertilizers to low benefit perception, lack of awareness, high input prices, poor credit markets, low farmer-return on investment or agronomic response^12,42^. Similarly, the failure of the recovery and reuse innovations to recuperate the costs is attributed to the poor understanding of the product markets^43–45^. The failure of composting innovations is mainly attributed to the low product demand^46,47^. The limited marketability and the bulkiness of compost are among other reasons for the low demand, making it costly to transport over long distances^47^. Although the technologies for recovering human excreta for agricultural use exist, scaling up such innovations would mainly depend on public acceptance of the end products^48^. Initiatives to address the poor demand include the fortification (agronomic response), pelletization/pelleting (visual appeal, handling, and bulk density), packaging (application instructions and nutrients content) and certification (risk perception). The current study, therefore, investigated the factors that influence the social acceptance of human excreta derived fertilizers in South Africa and the potential for wide-scale commercialization in rural communities. Investigating the factors influencing the market demand for compost could help to mitigate the stated failure of the recovery and reuse innovations through better understanding of the product market.

The current study is based on research gaps identified in a scoping review undertaken using the Preferred Reporting Items for Systematic reviews and Meta-Analyses (PRISMA). The objective of the study was to scope and synthesise the stock of published research on the social acceptance of human excreta reuse in agriculture. The findings demonstrate the paucity of published scientific knowledge on social acceptance of human excreta, and the inconclusive influence of demographic, sociological, and economic farmer characteristics on social acceptance^49^. It was impossible to draw meaningful conclusions from the small sample of published work. The review retrieved 22 published articles on the behavioral intentions to use human excreta. Fourteen peer-reviewed studies did not specify the recovery technology, while the remaining studies (*n* = 5) investigated human urine wastewater (*n* = 3), and composted faeces (*n* = 2). Research on the social acceptance of faecal sludge is, therefore, nascent and more studies are required to help inform profit-based business cases, research and development practice, and policymaking. The study findings could inform customer prospecting and segmentation, mainstreaming of policies and awareness campaigns, as well as targeting of innovative farmers to champion on-farm demonstration trials in development practice.

## Research methods

### Study area and design

A total of 341 farmers were interviewed in the Vulindlela Traditional Authority of South Africa, after obtaining a Humanities and Social Sciences Research Ethics Committee (HSSREC/00001499/2020) ethical approval and verbal consent from the Ethics Unit of the university’s Research Office (Figure S.1). The participants were provided with details of the study purpose, survey time, confidentiality, and the freedom to withdraw from the study at any time. An informed verbal consent was obtained from the participants before beginning the survey and the details of the informed consent are provided in the survey instrument, which is available at https://enketo.ona.io/x/#EkSVyazm. All the methods used in this study were performed in accordance with the guidelines and regulations of the Humanities and Social Sciences Research Ethics Committee. Power analysis was performed using at alpha = 0.05 and number of predictors of = 7, to detect a medium Cohen’s *d* effect size = 0.15, and the total sample size was calculated to be 153 with an actual power of 0.95. The G*Power software^50^ was used to determine the sample size and power calculations because of it is an open source software that is easy to use^51–53^. The sample size was however increased to the total sample size of 341 farmers, to accommodate two other studies that used the same survey instrument to collect information on ecological attitudes and willingness to pay using discrete choice experiments. The study adopted a cross-sectional study design which obtains information from all the respondents at a specific point in time. The detailed description of the study area, design, training, and budget is provided in the Supplementary Information S.1.

The survey data collection tool was developed using an XLS form, which was then converted to an XLM format for use in Open Data Kit mobile-based software solution. The instrument is available in ONA cloud server https://enketo.ona.io/x/#EkSVyazm. Online mobile-based survey tools allow for assigning of constraints and restrictions which helps to avert data collection and encoding errors, while increasing the data accuracy and reliability. The data is also available in an analysis-ready format that is compatible with the readily available software. The data collection tool was also tested online for content validity with academics, research, and development practitioners with operating within the food system and circular sanitation economy in agriculture projects. The detailed description of the nature of the survey questions is provided (S.3). The study used a multi-stage sampling procedure, to select two wards (ward 8 and 9) based on the maximum distance from the main city. A sampling interval was calculated to systematically select household units, where the main decision maker in the household was identified for interviewing. The non-response rate was negligible as absentee or inaccessible respondents were replaced by the closest house then resample from the newly selected household. The survey did not record any protesters nor failed to collect data because of the farmer’s refusal to participate in the survey. More details of the sampling strategy and the survey process are provided (S.4).

### Attitudinal dimension scores

All the data was managed and analysed using the IBM SPSS Statistics software package. The behavioral intentions of farmers to use human excreta was elicited using questions with the binary responses coded as 2 'yes' and 1 'no', such that the probability of the response was given as 1 ≤ μ ≤ 2^54,55^. The computation of the mean score (1 ≤ μ ≤ 2), was such that a mean score of 1.5 was considered neutral and a mean score greater than 1.5 indicate positive attitude, while below 1.5 suggests negative attitude. Some previous studies evaluated attitudes on a question-by-question basis^55^, and such methods are perfect for a more targeted understanding of specific questions of interest. However, it may be necessary to have several questions measuring a single construct to avert biases associated with single question responses. The responses were decomposed into a unidimensional construct to reduce the complexity of evaluating segregated attitudes on a question-by-question basis. This allows for mean comparison tests used to investigate the influence of different farmer-specific characteristics such as t-tests, ANOVA, and hierarchical regression, which depend primarily on the dependent variable's continuity in scale.

The *individual attitude score* was calculated by computing the mean scores of six attitudinal question items, namely; (i) willingness to use co-compost, (ii) willingness to use human urine, (iii) if the respondent thought human excreta should be disposed and never used, (iv) if the farmer would buy food produced using human excreta, (v) if the farmer would eat food produced using co-compost, and (vi) if the farmer would consume food produced using human urine. The attitude score was then segregated to reflect the production and consumption demand elements. The *production attitude* score was computed by taking the mean score of three out of the six attitudinal questions above, that are akin to production, namely: (i) willingness to use co-compost as a fertilizer, (ii) willingness to use human urine, (iii) if the farmer thought human excreta should be disposed and never used. The *consumption attitude score* was computed by taking the mean score of the three questions that relate to the willingness to consume and buy food produced using human excreta, namely: (i) willingness to buy food produced using co-compost (cost-risk element), (ii) if the farmer was willing to eat food produced using co-compost, and (iii) willingness to consume food produced using human urine.

The *perceived behavioral control score* was computed by taking the mean score of the four attitudinal question items that relate to self-efficacy and risk–benefit perception of using human excreta, namely: (i) if the farmer 'thought' that he/she had enough skills to use human excreta in farming, (ii) the effect of treatment on perceived health risk, (iii) whether the farmer thought that treated human excreta contains pathogens or microorganisms that can cause diseases, and (iv) whether the farmer thinks that pharmaceuticals/medicines can be found in crops grown with human excreta derived fertilizers. The attitudinal construct captures self-efficacy and perceived health risks.

Research in cognitive neuroscience demonstrates the existence of convergent human behavior^56^ and the co-influence of individual attitudes by the behavior of others^57^. The social cognitive theory posits that social learning occurs by modelling the behavior of other people or social conditioning from direct relational experience^58^. The *subjective norms score* was therefore computed by taking the mean score of the four question items that evaluate the influence of the behavior of others, namely: (i) do you think other people in general would use human excreta in their fields to fertilize crops? (ii) Do you think other people in the market would buy food produced using co-compost as fertiliser? (iii) Do you think your family members would eat food that was fertilised with human excreta? (iv) Do you think your neighbours, friends, relatives or other people would eat food that was fertilised with human excreta? The *combined attitude score* was then calculated by taking the mean score of all the computed constructs, namely, attitude score, perceived behavioral control and subjective norms.

### Environmental worldviews using the New Environmental Paradigm

The New (or Revised) Environmental Paradigm (NEP) responses were coded as 5-point Likert scale type questions, with 1 indicating strong disagreement while 5 represents strong agreement with the statement^59^. The seven even-numbered NEP statements where disagreement with the statements reflects a proenvironmental worldview were reverse coded following^55^. The overall NEP rating (1 ≤ μ ≤ 5) indicated the mean scores of all responses with 3 being neutral, 1 being extreme environmentally unfriendly, and 5 representing extreme proenvironmental or eco-friendly worldviews^60^. The current study used the Cronbach's Alpha (α) to test for the responses of the participants for internal consistency and reliability to the NEP statements. Although there are no absolute cut-off points for internal consistency, most research points to a minimum acceptable value of 0.70^61,62^. Other cut-off points that have been suggested in the literature for reliability analysis, include low reliability (< 0.50), moderate reliability, (0.50 ≤ α ≤ 0.70), high reliability (0.70 ≤ α ≤ 0.90), and excellent reliability (> 0.90)^62,63^.

### The direction and magnitude of the influence of demographic, socioeconomic and environmental factors on behavioral intentions

The objective of this study is to estimate the influence ex-ante the sociological, demographic, and socio-economic factors that influence and characterises the behavioral intention of farmers to use human excreta in agriculture. A family of hierarchical regression models were estimated using the 'naïve approach', where the dependent variables include (a) the individual attitude score, (b) the production attitude score, (c) the consumption attitude score, (d) the perceived behavioral control, (e) the subjective norms, and (f) the combined attitude score. The hierarchical regression approach is commonly used in the disciplines of psychology, sociology, and education to evaluate the incremental validity of the variables of interest, based on theory, past research, and the depth of understanding of the research problem^64^. The hierarchical regression approach is also the most used analytical approach for understanding the theory of planned behavior^65^. The predictive modelling approach is applicable in social science research, where independent variables are likely to be correlated and because of its superiority to the stepwise regression in terms of degrees of freedom, pre-specification effect, replicability, and sampling error^64^. Classical diagnostic tests did not show any violation of the classical regression assumptions. The income variable, which is normally used as an dependent variable is some cases was checked for endogeneity using the control function approach or the exclusion restriction method^66^. The method tests the suspected endogenous variable for potential influence on the dependent variable then uses this exclusion restriction to control for endogeneity in a two-step approach.

**Figure 1:**
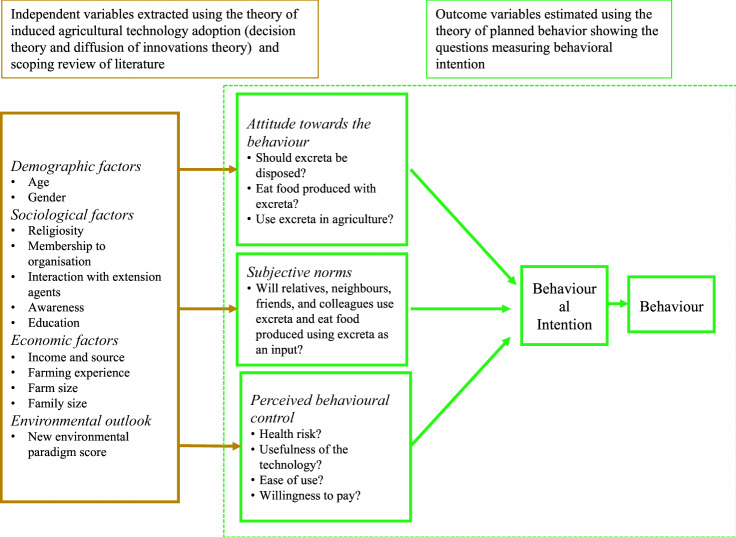
A Diagram of factors that influence behavioral intention to recycle human excreta in agriculture: a conceptual framework.

Although research organisations, governments, and development practitioners promote agricultural technologies, their adoption remains low. A meta-regression analysis of the adoption of agricultural technologies showed that, on average, education, family size, access to credit, land tenure and size, extension, and membership to farmer organizations have positive influence on the adoption of agricultural technologies^67^. Agricultural technology adoption theory combines decision theory and diffusion of innovations theory to understand the factors that influence farmers to adopt new technologies, leading to three paradigms: the perceptions, innovation-diffusion and the economic paradigms^67^. Diffusion is a social process of the spread of new innovations in the society over time^68^. Identifying the factors that influence decision or intention to adopt an innovation in the target population maximizes the diffusion efficiency initially focussing on innovators to champion on-farm pilot demonstration trials, while enhancing the design and implementation of awareness campaigns and dissemination plans^68^. Identification of the farmer characteristics may help to circumvent potential barriers before introducing the innovation into the community. The innovation-diffusion paradigm posits that information is the key driver of the diffusion of innovation and groups the adopters into innovators, early adopters, and laggards based on the observable demographic and socioeconomic attributes^69^. The economic constraints or utility-based paradigm and the adopter-perception paradigm posits that perceived attributes of innovations and innovators (farmer-characteristics) influence the perception (knowledge, attitudes and perceptions) that drives that diffusion process^70^. The conceptual and theoretical diagram describes how the agricultural technology adoption theory, and the scoping review of literature informed the identification of the factors influencing farmers’ behavioral intentions to recycle human excreta. The theory of planned behavior was used to identify the outcome variables, namely, attitudes, perceived behavioral norms and perceived behavioral control (Fig. 1).

## Results

### General characteristics of the interviewed farmers in Vulindlela rural traditional community

Reporting the demographic and socioeconomic characteristics of the study participants helps to understand the segregated attitudes of the farmers for customer segmentation. Approximately 68.2% of the 341 interviewed farmers were female, while 31.4% were male (Table S.4). The average age of the interviewed farmers, who were essentially household heads as described in the methods section, was 54 (14.2) years. The average and median (mean = median) years of education were approximately 8.0 (4.1) years (i.e., first year of secondary education), while the average farming experience in the area was 23.2 (3.3) years. The average number of household members in the traditional community was 6.3 members (Table S.5). Most of the farmers were married making up 43.7% of the households. The rest of the farmers were either single (32.0%), widowed (22.3%), or divorced (2.1%). In terms of religiosity, Christianity was the most popular religion making up 50.1% of the respondents, followed by polytheism (23.4%), traditionalism (12.6%), Shembe or Nazareth Baptist Church (7.9%), with atheists and agnostics making up the remaining 5%.

The study results show that 34.6% of households earn less than R12,000 per annum, 31.4% earn between R12,000 and R60,000, 18.2% between R60,000 and R100,000, while the remaining 15% earn greater than R150,000 per year (average exchange rate 1USD ≈ R15). Most of the income came from social grants (child support and old age), making up 60.7% of the households. The other sources of income were formal salary work (10.9%), casual labour (7.6%), remittances (6.2%), wage work (4.4%), sale of farm produce (3.8%), formal business (3.7%), informal economy (2.6%), and gifts (0.6%). Most of the farmers had smaller plots, with 77.4% of the participants owning less than a hectare of farming land. Although, all land belongs to the King, ownership in this study was based on the ‘permission to occupy’ type of lease, which guarantees the right to use the land. The tenure system does not give freedom to alienate, limiting the use of the land as collateral when performing economic and financial transactions. This type of lease may also limit investment in long-term benefits to the soil. The rest of the farmers were such that 19.6% owned between one and two hectares, while 3% owned more than two hectares. About 8.5% of the farmers belonged to some farming association, while 93% had never interacted with extension officers (Table S.5).

### General attitudes of farmers towards human excreta use in agriculture

The general attitude score was positive (1.62), indicating that farmers were willing to use human excreta-based fertilizers (Table 1). The estimated production attitude score was positive (1.59), while the consumption attitude score shows even more optimistic attitudes (1.66). The subjective norms were not as deterring as indicated by the generally positive (1.59) that others would comply with using, eating, and buying crops produced with human excreta. Perceived behavioral control presented potential barriers (1.43), indicating that farmers perceived lack of capability and some health risks in using human excreta in agriculture.

**Table 1:** Descriptive statistics of the attitudinal dimensions.

Attitudinal dimensions	Number of respondents	Mean scores	Standard deviation	Minimum	Maximum
Perceived behavioral control	341	1.43	0.23	1.00	2.00
Subjective norms	341	1.59	0.39	1.00	2.00
Overall attitude score	341	1.62	0.35	1.00	2.00
Production attitude score	341	1.59	0.36	1.00	2.00
Marketing attitude score	340	1.66	0.41	1.00	2.00
Combined attitude score	341	1.51	0.23	1.00	2.00

The farmers’ attitudes were mostly positive that treating human excreta would reduce risk with an average attitude score of 1.83. Farmers exhibited negative attitudes on questions relating to perceived behavioral control, including skills or self-efficacy, pathogen, and pharmaceutical risks, all of which had mean attitude scores below 1.50 (Table S.19). On average, farmers were highly positive on the use of human excreta-based co-compost (1.77). A mean score of 1.77 could also be interpreted as indicating that approximately 77% of the farmers agreed to recycling human excreta. Surprisingly, farmers were generally negative on using urine to fertilize their crops, with a mean attitude score of 1.48.

The mean market-related attitude scores indicated that farmers have a positive attitude on buying co-compost, and (1.73), buying food produced with urine (1.57). The analysis of subjective norms also indicates positive attitudes with farmers expecting that other people in general, would use human excreta (1.63), buy food produced using co-compost as fertiliser (1.71), and that other people would eat food fertilised with human excreta (1.59). In terms of whether human excreta should be disposed of, farmers were moderately positive, with mean attitudes score of 1.51 (Table S.19). Farmers expressed their doubt that other family members would eat food produced using human excreta with a sample mean attitude score of 1.42. The effect of crop type on willingness to accept human excreta was investigated in this study. Approximately 103 farmers (31%) of the sample farmers thought that the crop type fertilized with human excreta influenced their perceptions on the use of human excreta. Of the 103 farmers about 85% were willing to eat human excreta fertilised product if the fertilized crop was maize (Table S.10). A moderate 52% would eat vegetables, while only 41% were willing to eat root or tuber crops fertilized with human excreta.

### The direction and magnitude of the influence of demographic, socioeconomic and environmental factors on attitudes

**Figure 2:**
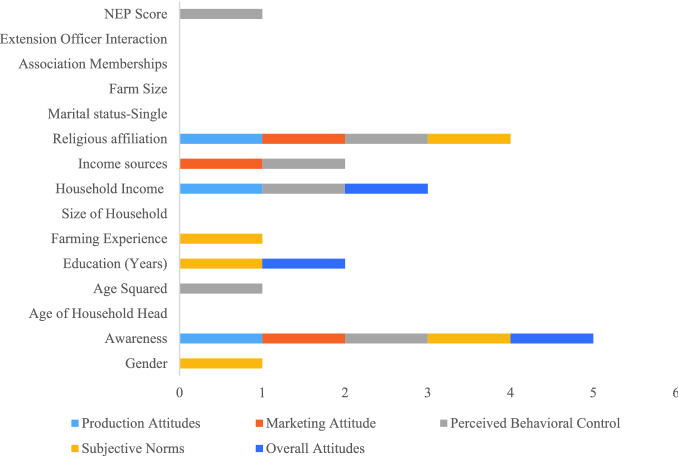
Segregated attitudes: the sensitivity of different attitudinal constructs to farmer characteristics.

The hierarchical regression models estimated using the 'naïve approach’ confirmed the importance of awareness in influencing social acceptance of human excreta. The results showed that awareness was the only independent variable that affects all dimensions of attitudes (Fig. 2). A unit increase in attitudinal scores explains the degree to which attitudes shift into positive (or negative) attitude by a unit change in the independent variable. For a positive shift in awareness, the average increase in all the mean attitudinal scores ranged between a moderate 0.06 units for perceived behavioral norms and as high as 0.24 (0.03) units for consumption-market related attitudes (Table 2). Religiosity was the second most variable regressor across the five attitudinal scores, namely, the production attitude score, the consumption-market related attitude score, the perceived behavioral control, and the subjective norms all increasing by orders of magnitudes of 0.13 at the least, and 0.22 units at most, for a unit change from Christianity to other religions.

**Table 2:** Segregated attitudes using the naïve regression approach.

Dependent variables	Production attitudes		Marketing attitude		Perceived behavioral control		Subjective norms		Overall attitude score	
Independent variables	Coefficient	Std error	Coefficient	Std error	Coefficient	Std error	Coefficient	Std error	Coefficient	Std error
Gender	− 0.015	(0.04)	0.041	(0.05)	− 0.020	(0.03)	0.109**	(0.05)	0.045	(0.03)
Awareness	0.234***	(0.04)	0.241***	(0.05)	0.060**	(0.03)	0.148***	(0.05)	0.104***	(0.03)
Age of household head	− 0.002	(0.01)	0.005	(0.01)	− 0.010	(0.01)	0.012	(0.01)	0.001	(0.01)
Age squared	0.000	(0.00)	− 0.000	(0.00)	0.000*	(0.00)	− 0.000	(0.00)	− 0.000	(0.00)
Education (years)	0.011	(0.01)	0.008	(0.01)	0.003	(0.00)	0.018**	(0.01)	0.010**	(0.00)
Farming experience	0.002	(0.00)	− 0.002	(0.00)	− 0.001	(0.00)	0.004*	(0.00)	0.001	(0.00)
Size of household	0.000	(0.01)	0.005	(0.01)	− 0.001	(0.00)	− 0.000	(0.01)	− 0.001	(0.00)
**Household income less than R12 000**										
R12 000 ≤ years < R30 000	0.043	(0.05)	− 0.083	(0.06)	− 0.010	(0.04)	− 0.003	(0.06)	− 0.007	(0.04)
R30 000 ≤ years < R60 000	0.070	(0.08)	− 0.088	(0.09)	− 0.019	(0.05)	− 0.031	(0.09)	− 0.025	(0.05)
R60 000 ≤ years < R100 000	0.145*	(0.07)	0.026	(0.09)	− 0.039	(0.05)	− 0.104	(0.08)	− 0.071	(0.05)
R100 000 ≤ years < R150 000	− 0.011	(0.08)	− 0.128	(0.09)	− 0.068	(0.05)	− 0.066	(0.09)	− 0.067	(0.05)
Greater than R150 000	− 0.074	(0.12)	− 0.120	(0.14)	− 0.193**	(0.08)	− 0.099	(0.14)	− 0.146*	(0.08)
**Income sources-farm or agricultural sales**										
Formal salary work	0.063	(0.11)	0.235*	(0.13)	− 0.103	(0.08)	0.059	(0.12)	− 0.022	(0.07)
Informal economy	− 0.004	(0.11)	0.081	(0.13)	− 0.092	(0.07)	− 0.053	(0.12)	− 0.072	(0.07)
Remittances/gifts	0.094	(0.12)	0.232	(0.15)	− 0.155*	(0.08)	0.108	(0.14)	− 0.023	(0.08)
Social grant	− 0.041	(0.10)	0.140	(0.13)	− 0.128*	(0.07)	0.030	(0.11)	− 0.049	(0.07)
**Christianity-religious affiliation**										
Traditionalism	0.035	(0.05)	− 0.023	(0.06)	0.029	(0.04)	− 0.022	(0.06)	0.004	(0.03)
Polytheism	0.103	(0.14)	0.010	(0.16)	0.216**	(0.10)	0.021	(0.16)	0.118	(0.09)
Shembe	0.130**	(0.06)	0.167**	(0.07)	− 0.038	(0.04)	0.136*	(0.07)	0.049	(0.04)
**Single-marital status**										
Married	− 0.000	(0.06)	0.033	(0.07)	0.021	(0.04)	− 0.097	(0.07)	− 0.038	(0.04)
Divorced	− 0.021	(0.04)	− 0.026	(0.05)	− 0.005	(0.03)	− 0.060	(0.05)	− 0.032	(0.03)
Widowed	0.019	(0.07)	0.052	(0.09)	0.031	(0.05)	0.040	(0.08)	0.036	(0.05)
Farm Size	0.045	(0.05)	− 0.004	(0.06)	0.023	(0.03)	− 0.033	(0.05)	− 0.005	(0.03)
Association Memberships	0.113	(0.07)	− 0.003	(0.08)	− 0.066	(0.05)	− 0.045	(0.08)	− 0.056	(0.05)
Extension interaction	0.047	(0.08)	0.006	(0.10)	0.054	(0.06)	− 0.153	(0.09)	− 0.049	(0.06)
NEP score	− 0.020	(0.04)	− 0.020	(0.05)	0.067**	(0.03)	− 0.028	(0.05)	0.020	(0.03)
Constant	1.234***	(0.32)	1.095***	(0.38)	1.536***	(0.22)	1.025***	(0.35)	1.280***	(0.21)
R-squared	0.233		0.184		0.116		0.175		0.142	
Degrees of freedom	310		309		310		310		310	
BIC	346.4		456.1		99.4		423.4		75.3	

*p < 0.1, **p < 0.05, ***p < 0.01.

The third most sensitive variable was income. A change in the income from the lower to the middle-income group had a 0.15 increase in production attitudes. However, if income was to increase beyond the middle-income group to greater than R150 000 per year, farmers' attitudes were reduced by 0.19 units and 0.15 units for subjective norms and overall attitudes, respectively. A ten-year increase in education improves subjective norms by 0.20 score points and the overall attitude score by 0.10 units, confirming education's importance in changing subjective norms and overall attitudes. A 10-year increase in farming experience results in a 0.04-unit change in subjective norms score. Changing the income source from agricultural sales to remittances and social grants reduced perceived behavioral control by 0.13 and 0.16, respectively. Relying on agriculture to eke a living increased social acceptance. Being male also had a positive impact on subjective norms by 0.11 units. In terms of the environmental outlook, a positive unit increase in the NEP score had a 0.07 unit increase in the subjective norms. The results suggest that being proecological increases the social acceptance.

## Discussion

### General attitudes of farmers towards human excreta

This study explored farmers' recycling behavioral intentions in the traditional and rural community of Vulindlela in the KwaZulu-Natal province of South Africa, by employing the theory of planned behavior as a conceptual framework. The attitudes towards human excreta use in agriculture were highly positive. The farmers also showed moderate positive environmental attitudes with a mean attitude score, which significantly differed across the perceived behavioral control and subjective norms. Environmental consciousness was positively related to willingness to recycle human excreta. The influence of proenvironmental attitudes on human excreta confirm the results reported elsewhere^71^. Environmental consciousness was found to influence the willingness to adopt urine-diverting toilets and human waste recycling in Hawaii^54^. Using university participants^55^, reported a different result, where environmental attitudes did not influence urine recycling attitudes. The different contexts (India vs. South Africa/Hawaii), study participants (farmers vs. university students), human excreta product (urine vs. urine and co-compost), and research methods may explain the different outcomes observed in the two studies.

This study has already confirmed differences in farmer preferences between urine and human faeces, with the latter being more preferred than urine. The result can be explained by the fact that farmers could be willing to work with products that they are most familiar with since co-compost is similar in attributes to ordinary compost or livestock manure. The study findings also validate the result that 70% of the farmers use compost-like material as soil conditioners namely, cow manure, poultry manure, organic compost, and farm residues. This result confirms the findings from US farmers where the perceived benefits and social acceptance was higher for biosolid-based fertilizers when compared to human urine-derived fertilizers^48^. This study also indicated that farmers were generally willing to use human excreta-based fertilizers, suggesting presence of a potential demand or social acceptance. The estimated production attitude consumption attitude score, score, and the subjective norms indicated that farmers were willing to use, buy, and consume products produced using human excreta. Perceived benefits may positively influence attitudes towards co-compost reuse. Empirical evidence shows that perceived benefits may include economic benefits^71,72^, and soil health improvement^72–77^. Co-compost application could also enhance crop-fertilizer response thereby supplementing and complementing chemical fertilizers^10,40,42^.

Results indicate that farmers reported negative perceived behavioral control which implies perceived health risk and low self-efficacy, or self-evaluation of skills required to use human excreta potential. The negative perceived behavioral control has been reported elsewhere where respondents were less confident of urine recycling due to perceived pathogen risk and pharmaceuticals^78^. Health risk perception has been reported in several studies as the main barrier to human excreta reuse in agriculture^75–77,79–81^. The perceived health risks span from the awful smell, skin infections, and other occupational hazards^82^. Socio-cultural factors, norms, religion, and taboos were also found to be barriers to human excreta use in previous studies^71,83–88^. On average, the farmers were moderately positive on whether human excreta should be disposed, confirming their moderate environmental consciousness. However, the farmers expressed doubt on whether family members would eat food produced using human excreta, indicating strong influence of subjective norms.

### The implications of attitudes of farmers towards human excreta for policy, and development practice

A total of ten variables significantly influenced the attitudinal dimensions. These include awareness, religion, education, age, interaction with extension officer, environmental consciousness, gender, farming experience, income, and source of income. Awareness, religiosity, education, and environmental consciousness positively influenced overall attitudes. The findings of this study fortify the significance of mainstreaming context-specific dissemination strategies in circular nutrient economy initiatives. Farmer awareness of human excreta reuse was the most important factor. Early studies confirm the positive relationship between awareness and recycling behavioral intention^89^. The effect of awareness on attitudes also confirms the reported positive influence of mass media communication on subjective norms^54^. Six studies reported the lack of awareness as a perceived barrier to human excreta reuse in farming^74,77,79,80,90,91^. Awareness and education were reported elsewhere to reduce the perceived health risks^92^. This study therefore demonstrates that raising awareness and educating farmers could improve self-confidence and reduce perceived health risks. The level of education has been previously reported to have a positive effect on the attitude towards human excreta recycling^81,86,93^.

Religiosity was the second most variable regressor across the five attitudinal scores increasing for a unit change from Christianity to other religions. The result is surprisingly paradoxical given the importance of ecological sanitation in the Deuteronomic code of the Biblical text. In Deuteronomy 23:10–15, the Israelites were instructed by God in the Deuteronomic laws (chapters 12–26) to practice complete burial of human excreta on the ground, which has been linked to the concept of ecological sanitation by theologians and ethicists who are shifting their understanding to tracing the roots of environmental concerns in biblical sources^94^. The positive link between religious education and environmental attitudes is also well established in the literature^95^. However, sensitivity to religious view is important for customer prospecting, and the designing and implementation of dissemination strategies.

Other variables such as age, farming experience, income, and income source were found to negatively influence subjective norms and consumption-related attitudes. Five variables, namely, household size, marital status, farm size, membership to an association, and extension officer interaction did not influence behavioral intentions. Younger farmers were more positive towards reusing human excreta. The result confirmed the findings from two other studies in South Africa, which indicated that younger farmers had more positive attitudes^79,96^. These results contradicted with findings in India, where older farmers expressed a more positive attitude^72^. The results also indicate that the more experienced farmers are in agriculture are more comfortable with their current technologies, and the less likely they accept new technologies. The negative income effect indicated that lower-income farmers were more willing to use human excreta, validating the results reported in other studies^97,98^.

Targeting low-income groups as champions in the demonstration of circular bioeconomy innovations could guarantee the social acceptance. The older farmers and middle-income groups who source their income from non-agricultural sources (such as social grants and casual labour) could only adopt the innovation if they see it working with the innovators. The influence of socioeconomic farmer characteristics, such as experience in farming, income, farm size, and agronomic benefits on the attitude towards human excreta reuse in agriculture, has been reported in other studies^97,98^. Male farmers perceived that negative influence of subjective norms. Female farmers showed a negative attitude compared to male farmers as reported in^86^, and male farmers had a positive attitude towards eating human excreta fertilized food compared to female farmers^73^.

There is substantial evidence indicating the significance of addressing the local context for the exploitation of the potential of new information and communication technologies in developing countries^99^. Policymakers could integrate the circular bioeconomy ideas based on indigenous knowledge systems to add value to the development policy practice while shifting the existing paradigms. Policy incentives for co-composting could include viability gap funding, clean development mechanisms, fair competition from mineral fertilizers, and an enabling regulatory environment for circular bioeconomy approaches. The public institutions, private sector, non-government organizations, and co-operative development partners could provide the capital required for setting up scalable, and viable co-composting projects. The donor support could help absorb the start-up costs such as awareness building, activating demand, and technical training^100^.

A transdisciplinary dissemination approach that appreciates human excreta reuse as more than a technology, but an innovation process operating within a socio-technical system, could help to co-design and co-develop farmer-driven dissemination plans and marketing strategies. The study results could enhance market segmentation and prospecting of innovative farmers to champion the on-farm testing and piloting of scalable co-compost innovations. The use of the World Health Organisation sanitation safety-planning manual as a template for developing local training material could lower the perceived risks and low self-efficacy. Awareness campaigns could enhance self-confidence by the end-users to maintain the technologies and to use the products^101^. This could be enhanced by early community involvement through assessing the possible products needed from FS and willingness to produce and use these products^102^. Demonstration projects are among the most effective marketing strategies used to penetrate new markets^47^. A sustainable systemic wide-scale adoption of innovations is only possible through the public and the private sector, which can sustain the change over time^103^. Scaling occurs when there is sustained and systemic change to a new normal beyond the funded project’s time frame^104^.

### Other driving forces and potential barriers and future research direction

The effect of crop type, processing, and cooking influenced farmers’ perceptions on the use of human excreta. South Africa's experiences with domestic treated wastewater effluent show the importance of choosing the right crops by avoiding crops that are consumed raw, while prioritizing crops with edible parts wrapped in husks, pods, and peels^105^. Moving away from crops such as cucumbers, carrots, and lettuce towards maize and beans may enhance social acceptance. The effect of treating human excreta, crop type, processing, and cooking food on farmers' attitudes was not investigated in the literature. The results of this study therefore provide a preliminary insight to these issues and create an interesting point of departure for in-depth future studies. The study findings show the importance of treatment and certification, processing, and cooking in promoting the use of human excreta in agriculture.

The results from the research from the social acceptance of genetically modified and organic foods reinforce the influence of processing on consumer willingness to accept^106–110^. The further the distance of human excreta-derived fertilizers from the consumer, and change in form through processing, the more likely farmers are willing to consume the food produced from it. Processing tomatoes into tomato sauce, maize into instant porridge, or sugarcane into table sugar may enhance social acceptance. Our findings show that most farmers in rural South Africa choose their main fertilizers based on availability, price, safety, and certification. Empirical evidence shows that availability, transport, storage costs, and perceived self-efficacy are potential barriers to reusing human excreta in agriculture^83,85,97^.

Other desirable characteristics investigated in this study include the nutrient content, packaging, credit facilities, pelletization, and recommendation by trusted sources (Table S.9). Specific to co-compost, the most reported desirable characteristics include soil health productivity, treatment, and the fact that it enables farmers to buy fewer chemical fertilizers. The importance of providing compost in the right attributes to farmers and the cost of providing such attributes have been investigated elsewhere although it remains a nascent and an important area for future research^111^. The farmers who were not willing to use co-compost reported smell, current use of chemical fertilizer, fear of being mocked, the need for more research, health risk, organoleptic/tastes, disgust, and religions or taboos as the potential reasons for resistance to using human excreta in agriculture (Tables S.13 and S.14). For instance, findings from the US suggest that disgust is not a major driving force to acceptance^112^. While most of these elements can be addressed by raising awareness, hygiene practices, and education, the health risk perception are of technical concern for contaminant elimination. Using the World Health Organization Sanitation Safety Plans to perform microbial risk assessment across the human excreta recovery, and reuse chain is recommended for protecting farmers' health^105^.

## Limitations

Some exciting elements were not explored to reduce the length of the paper. The environmental attitudes could have been explored further, primarily, the effects of demographic, socioeconomic, and cultural factors, and the nature of the ecological dispositions of rural farmers. Another interesting dimension would be to block the study by different environments so that attitudes can be evaluated for farmers in rural and urban settings and compare the results for a more targeted development approach. The latent structure and the dimensionality of the NEP scale against the five worldviews: (1) reality of limits to growth, (2) antianthropocentrism, (3) fragility of nature's balance, (4) rejection of human exemptionalism (the belief that humans are exempt from environmental forces), and (5) the possibility of an ecocrisis was not evaluated in this study. The issues may provide an interesting area for future research.

## Conclusion

Behavioral intentions to use human excreta were evaluated using the theory of planned behavior in this study. The segregated attitudes were evaluated for production attitudes, consumption attitudes, subjective norms, perceived behavioral control, and the overall or combined attitudes. Attitudes towards the reuse of human excreta are mainly sensitive to subjective norms and perceived behavioral control suggesting the importance of understanding local context when mainstreaming recycling initiatives and when designing and implementing dissemination plans and strategies. The findings of this study suggest that there is demand for human excreta derived fertilizers in rural agricultural communities of South Africa. The farmers exhibited positive attitude towards the recycling of human excreta in agricultural food systems.

The effect of farmer characteristics, such as religiosity, income, education level, gender, and environmental consciousness need to be understood and tailor interventions and target customer segments, rather than implementing blanket recommendations. The perceived behavioral control was reported to be a potential barrier to human excreta reuse in agriculture, indicating strong influence of health risk perception and demand for skills. Behavioral intentions to use human excreta were driven by age, awareness, religiosity, income, income source, and environmental disposition. Understanding the nature, and direction of the influence of attitudinal dimensions and farmer characteristics is important for mainstreaming circular bioeconomy interventions.

## Supplementary Information

Supplementary Information.

## Data Availability

The raw data and collated data supporting the findings of this study could be made available from the corresponding author upon judicious request.

## Code availability

The computer code supporting the findings of this study could be made available from the corresponding author upon judicious request.
