# Supplementary material for: Behavioral intentions of rural farmers to recycle human excreta in agriculture
Source: Sci Rep. 2022 Apr 7;12:5890. doi: 10.1038/s41598-022-09917-z (PMC8989988; doi:10.1038/s41598-022-09917-z)
Supplement: Supplementary file 1 — Supplementary Information. [file 41598_2022_9917_MOESM1_ESM.docx]

**Behavioral intentions of rural farmers to recycle human excreta in agriculture**

**Supplementary Information**

**Simon Gwara^1^*, Edilegnaw Wale^2^, and Alfred Odindo^3^**

^1^ Discipline of Agricultural Economics, School of Agricultural, Earth and Environmental Sciences, University of KwaZulu-Natal, Pietermaritzburg, 3201, South Africa

^2^ Department of Agricultural Economics, Faculty of Natural and Agricultural Sciences, University of the Free State, Bloemfontein, 9300, South Africa; zegeyeew@ufs.ac.za

^3^ Discipline of Crop Science, School of Agricultural, Earth and Environmental Sciences, University of KwaZulu-Natal, Pietermaritzburg, 3201, South Africa; odindoa@ukzn.ac.za

***** Correspondence: 218086735@stu.ukzn.ac.za or [simmonsgwara@yahoo.co.uk](mailto:simmonsgwara@yahoo.co.uk)

This appendix contains all the detailed information that could not be included in the main text as it would distract the reader from the main theme. The supplementary information soft-copy information such as specific details of statistical analyses, the results of questionnaires, spreadsheets of data, and the other materials reference in the main in the main chapters.

*S.1: Detailed study area description*

Vulindlela Traditional Council consists of nine wards under the Ingonyama Trust Board administration and the sole trustee of His Majesty the King (Isilo/Ingonyama) Goodwill Zwelithini (Kharsany et al., 2015; Msunduzi Municipality, 2016). The ten wards include wards 1 to 9 (Piper and Deacon, 2008) and recently incorporated ward 39 (**Table S.1 and Table S.2**). The area occupies 40% of the Msunduzi Municipality, covering approximately 25 000 hectares. The Vulindlela area accommodates more than 85 000 households and approximately 150 000 predominantly IsiZulu-speaking population (Kharsany et al., 2015). The land-use patterns include a mix of dwellings, grazing areas, individual farmlands, community gardens, indigenous forests, and timber plantations. The structures within the Vulindlela consists of the traditional structures (Amakhosi-chiefs and Izinduna-chief's headmen), political structures including community development structures and municipal councilors, government departments, para-state structures in energy and water, and other non-governmental organisations (Chirowodza et al., 2009). The location of Vulindlela as a ‘gateway’ to the city (25-40km from the city center) is of importance for building a resilient city-region food system. Rural and peri-urban agriculture contributes to a more diversified food basket and provides access to fresh perishable foods while generating income and employment, not only for farmers and farm labourers, but through multiplier effects in the economy. Peri-urban agriculture helps to save economic and environmental costs of transportation and cooling facilities for perishable food commodities (Hofny-Collins, 2006).

*S.2: Detailed study design, survey training, and budget*

We adopted a cross-sectional study design for this study which obtains all the respondents' measurements at a specific point in time. In this study, each farmer was interviewed once, although different households were interviewed on different dates. The household survey (preparation, training, and interviews) was administered from 10 to 26 November 2021 during a national lockdown and university Covid-19 window where household surveys were temporarily allowed with the actual fieldwork taking less than ten days. The enumerators were always encouraged to adhere to the country's Covid-19 lockdown level 1 regulation. The hiring of enumerators for training was advertised within the School of Agriculture, Earth and Environmental Sciences, requiring enumerators with minimum qualifications of being an enrolled master's degree student with exceptional skills of the IsiZulu language. Hiring qualified students came at a higher cost because universities use stipulated daily remuneration guides for fixed-term appointments. The rough order of magnitude estimates increased upwards by R60 000 (average exchange rate at the time 1USD ≈ R15), including the costs of car hire, enumerators, field guides, and electronic gadgets (**Table S.3**).

A two-day training was implemented to ensure validity, identify unforeseen and avertable problems, and improve data quality and accuracy. The study, therefore, required enumerators with exceptional translation skills and a high level of conceptualisation, including the ability to make sensible conversations with rural farmers. The questions that required exceptional skills from our experience include the New Ecological Paradigm (NEP) and the attitudinal questions. The field supervisor was a doctoral student in agricultural economics. The training covered survey methodology, review of mobile-based cloud data collection methods, the survey instrument, fieldwork principles, and interview hints and tips. The accuracy of the data can be assumed to be moderately high, considering the investment in training and recruiting qualified enumerators.

*S.3: Survey questions*

The survey mostly included single response and closed-ended questions that were either binary (Yes /No) or multiple-choice type after seeking participant consent and ethical approval (Fig S3). A choice was made between the two types of questions commonly used in cross-sectional surveys: open-ended and closed-ended questions. Closed-ended questions were used mostly to reduce the respondents' cognitive burden in trying to respond with an explanation to issues they may not be knowledgeable about. This explains why the selection of closed-ended questions in studies with smaller samples or populations is preferred, which treat each response as a unique opinion. For instance, only a few cases, 'other reason(s)', were open-ended where the respondents were given an opportunity to give their opinion other than those defined by the researcher. Although respondent answers from open-ended qualitative questions almost always provide a richer quality data, it can be unwieldy to make useful conclusions from the data. Demographic and socioeconomic questions such as the age, education, farm experience, income, income sources, gender, and religious affiliation, interaction with extension, farm size, and family size of the household head were also collected. Other question types included a 5-point Likert scale type question, for instance, in eliciting the New Ecological Paradigm (NEP) scale to assess environmental attitudes.

*S.4: Sampling strategy*

The study used a multi-stage sampling procedure, which may not be as effective as the true random sampling but may solve the challenges inherent in random sampling, which is used when a complete list of all members of the population is available. The use of multistage sampling averts the large, and perhaps unnecessary, costs associated with traditional cluster sampling by not using all sample units in all selected clusters. A multistage procedure was implemented by first purposively selecting 2 wards, randomly selecting villages and households in each ward. The selected wards (ward 8 and 9) were based on the maximum distance from the city. The number of households and people within each of the 10 wards that make up the Vulindlela Traditional Authority was accessed from the online data available at <https://wazimap.co.za> (**Table S.2)**.

For this study, the sampling unit - a household - was defined as people living together for more than three months, eating from the same pot, and making important livelihood or food security decisions together. The household head was the primary decision-maker on farming activities and not necessarily the head in the traditional/cultural/contextual sense. Initially, the sampling interval - the space between each selected household - was calculated by dividing the total number of households in each ward by the sample size. However, we saw that the sampling interval was creating large distances for enumerators to walk between houses, including the high non-response rates, the selection of households was therefore done by dropping enumerators into clusters where they would select five households. The relative homogeneity of the farmers in the community should not significantly affect the randomness of the sample. A neighbouring farmer was selected in the event of non-response or absence of the selected farmer.

**Table S.1** Traditional Councils in Vulindlela

| Traditional Councils in in Vulindlela Community (25 209.68ha) | Wards in each Traditional Council | The Inkosi/Leadership |
| --- | --- | --- |
| Mpumuza Traditional Council | 1, 2, 8 | Inkosi N.W Zondi |
| Inadi Traditional Council | 3, 4, 5, 9 | Inkosi SG Zondi |
| Mafunze Traditional Council | 7, 39 | Inkosi MSP Ngcobo |
| Ximba Traditional Council | 6 | Inkosi S Malaba |
| Nxamalala Traditional Council | 3 | Inkosi E.S Zuma |

**Table S.2.** Sampling information for Vulindlela Tradional Council

| Wards-in Vulindlela Community (25 209.68ha) | Estimated enumeration areas or villages *(isigodis)* in each ward | Names of the smallest sampling units (Isigodis/EAs) for which data is available | Estimated population in sampling unit | Estimated household units in each sampling unit | Population in each ward | Households in each ward |
| --- | --- | --- | --- | --- | --- | --- |
| 1 | 1 | Mpumuza/*Phayapini* | 11668 | 2678 | 18587 | 4043 |
| 2 | 2 | Zayeka/Mthoqotho | 8762+6699=15461 | +1791+1387=3178 | 17444 | 3604 |
| 3 | 4 | KwaMpande/Mgwagwa/Kwadlulela/Ebaleni | 6710+2135+1766+873=11484 | 1201+413+323+148=2085 | 16909 | 3361 |
| 4 | 1 | Eshowe/Esimbovu | 1182 | 236 | 11239 | 2314 |
| 5 | 2 | Gezubuso/Noshezi | 5656+777=6433 | 1179+156=1335 | 17040 | 3761 |
| 6 | 2 | Qanda/Etafuleni/Ntembeni | 2164+1104=3268 | 379+238+=617 | 15236 | 2836 |
| 7 | 4 | Embabane/Enzondweni/eMunywini+eMunyini | 3118+1924+2320+394=7756 | 559+384+486+66=1495 | 14342 | 2717 |
| 8 | 2 | *Emaswazini*/Madladla/Elandskop/*Mcane*/ | 3669+10229=13898 | 713+1902 = 2615 | 11330 | 2145 |
| 9 | 1 | Mafakatini/Taylor’s Halt/ | 8085 | 1524 | 15540 | 2 971 |
| 39 | 1 | 39 | 13586 | 2 544 | 13586 | 2 544 |
| Total |  |  | 79 235 | 15 823 | 151253 | 24781 |

<https://census2011.adrianfrith.com/place/566>

**Table S.3.** First cut survey budget-including pilot testing and training

| Item |  | Unit cost [R/day] | No. of persons | Number of days | | Total Cost[R/day] |
| --- | --- | --- | --- | --- | --- | --- |
| Per diems | Supervisors | 500 | 1 | | 10 | 5 000 |
|  | Enumerators | 500 | 8 | | 10 | 40 000 |
| Allowances | Field assistants | 350 | 2 | | 10 | 7 000 |
| Car Hire |  | 700 | 2 | | 15 | 24 000 |
| Grand total |  |  |  | |  | 75 000 |

**Table S.4.** Characteristics of survey respondents (continuous scale)

| Household characteristic | Mean | Median | Max | Min | Standard Dev |
| --- | --- | --- | --- | --- | --- |
| Age (years) | 54 | 57 | 88 | 20 | 14.2 |
| Years of education | 7.9 | 8 | 19 | 0 | 4.1 |
| Farming experience | 23.2 | 20 | 70 | 1 | 15.6 |
| Household size | 6.3 | 6.0 | 17 | 1 | 3.3 |

**Table S.5.** Characteristics of survey respondents (nominal scale)

| Household characteristic | Percentage (%) | Frequency |
| --- | --- | --- |
| Gender | 100% | 341 |
| Female | 68.6 | 234 |
| Male | 31.4 | 107 |
| Marital Status | 100% | 341 |
| Married | 43.7 | 149 |
| Single | 32.0 | 109 |
| Widowed | 22.3 | 76 |
| Divorced | 2.1 | 7 |
| Religious affiliation/practice? | 100% | 341 |
| Christianity | 50.1 | 171 |
| Polytheism | 23.4 | 83 |
| Traditionalism | 12.6 | 43 |
| Shembe/Nazarene | 7.9 | 27 |
| Other | 2.6 | 9 |
| Atheism | 2.1 | 7 |
| Agnosticism | 0.3 | 1 |
| Annual Income | 100% | 341 |
| < R12 000 | 34.6 | 118 |
| R12 000≤ Y< R60 000 | 31.4 | 107 |
| R60 000≤ Y<R100 000 | 18.2 | 62 |
| Greater than R150 000 | 15.8 | 54 |
| Source of Income | 341 | 100 |
| Social grant | 60.7 | 207 |
| Formal salary work | 10.9 | 37 |
| Casual labour | 7.6 | 26 |
| Remittances | 6.2 | 21 |
| Wage work | 4.4 | 15 |
| Sale of farm produce | 3.8 | 13 |
| Formal business | 3.7 | 11 |
| Informal economy | 2.6 | 9 |
| Gifts | 0.6 |  |
| Farm Size | 100% | 341 |
| ≤ 1 ha | 77.4 | 264 |
| 1–2 ha | 19.6 | 67 |
| 3–4 ha | 1.8 | 6 |
| > 4 ha | 1.2 | 4 |
| Membership | 100% | 341 |
| Yes | 8.5 | 29 |
| No | 91.5 | 312 |
| Extension officer interaction | 100% | 341 |
| Never | 93.8 | 320 |
| Less than once a year | 2.6 | 9 |
| Once a year | 2.3 | 8 |
| At least twice a year | 0.3 | 1 |
| More than twice a year | 0.9 | 3 |

**Table S.6.** Main or dominant fertilizer in production system

| Fertilizer type | Frequency | Percentage (%) |
| --- | --- | --- |
| Inorganic fertilizer | 66 | 19.5 |
| Poultry manure | 22 | 6.5 |
| Cow manure | 201 | 59.3 |
| Organic compost | 13 | 3.8 |
| Co-compost | 5 | 1.5 |
| Farm residues | 10 | 2.9 |
| Others | 22 | 6.5 |
| Total | 339 | 100 |

**Table S.7.** Reasons for the dominant fertilizer

| Fertilizer type | Percentage (%) | Frequency |
| --- | --- | --- |
| Availability | 79.6 | 269 |
| Price | 3.6 | 12 |
| Environmentally friendly | 2.1 | 7 |
| Soil health | 14.8 | 50 |

*S.5. Fertilizer types, reasons for use, and sources*

The farmers' main type of fertilizer in the study area is cow manure (59,3%), followed by inorganic fertilizers, used by 19.5% of the respondents (Table S.6). Other types include poultry manure (6.5%), organic compost (3.8%), co-compost (1.5%), and farm residues (2.9%). A total of 22 farmers, constituting 6.5%, relied on other forms of fertilizers or did not use fertilizers at all in their farming systems (Table S.7). Most farmers in this community use cow manure, making it easy for farmers to accept similar products. Approximately 80% of the farmers chose their dominant fertilizer based on availability. Other reasons include soil health (14,8%), price (3.6%), and environmental benefits (2.1%). The main source of these fertilizers was free, making up 48.3% of the respondents, while 41.4% were producing it on the farm. The rest of the farmers bought their fertilizers (8.6%) or produced them elsewhere (1.4%) (Table S.8).

**Table S.8.** Sources fertilizer

| Source | Frequency | Percentage (%) |
| --- | --- | --- |
| Produce it on the farm | 58 | 41.4 |
| Produce it elsewhere | 2 | 1.4 |
| Buy it | 12 | 8.6 |
| Get it for free | 68 | 48.6 |
| Total | 140 | 100.0 |

**Table S.9.** Factors influencing consumers’ purchasing behaviour

| Statement | Level of agreement % | | | | |
| --- | --- | --- | --- | --- | --- |
| Desirable characteristic | Strongly Disagree | Disagree | Don’t know/neutral | Agree | Strongly Agree |
| Price | 10,6 | 9,1 | 8,2 | 39,4 | 32,6 |
| NPK content | 2.4 | 4.4 | 22.6 | 41.5 | 29.1 |
| Organic matter | 1.8 | 1.8 | 8.8 | 53.1 | 34.5 |
| Safety | 1,8 | 1,8 | 8,8 | 53,1 | 34,5 |
| Packaging | 8.9 | 22.6 | 19.6 | 33.2 | 15.7 |
| Certification | 5,6 | 7,6 | 14,1 | 38,8 | 33,8 |
| Credit offer | 7.1 | 15.7 | 24.6 | 39.6 | 13.0 |
| Convenient location | 5.0 | 10.0 | 12.9 | 48.2 | 23.8 |
| Pelletization | 8.3 | 17.1 | 22.7 | 26.5 | 25.4 |
| Recommended by sources I trust | 6.2 | 7.7 | 10.3 | 45.7 | 30.1 |

**Table S.10.** Effect of crop type on attitudinal score

| Attitude item | Frequency  (Yes) | Percentage (Yes) | Total number of responses |
| --- | --- | --- | --- |
| Does crop type to be fertilized with co-compost matter to you? | 103 | 30.8 | 334 |
| If crop type matters to you, would you eat leafy vegetables fertilized with co-compost? | 54 | 52.4 | 103 |
| If crop type matters to you, would you eat roots crops/tubers fertilized with co-compost? | 42 | 40.8 | 103 |
| If crop type matters to you, would you eat maize fertilized with co-compost? | 87 | 84.5 | 103 |

**Table S.11.** Factors that drive farmers to use co-compost on their crops

| Statement | Percentage | |
| --- | --- | --- |
|  | Yes | No |
| Co-compost is good for my soil | 96.2 | 3.8 |
| Co-compost will increase my crop productivity | 96.6 | 3.4 |
| Co-compost is good if it is sanitised and used safely | 88.8 | 11.2 |
| If I use co-compost, I must buy less fertiliser from the market; | 85.4 | 14.6 |

**Table S.12.** Any other reasons for using co-compost (post-coded)

| Any other reasons for using co-compost (post-coded) | Number of respondents |
| --- | --- |
| Appearance | 1 |
| Applies to more crops-promotes diversification | 7 |
| Availability | 3 |
| Fertility | 1 |
| Good yields | 42 |
| Lower price | 6 |
| Safety | 3 |
| Sanitation improvement | 12 |
| Sustainable | 2 |
| Uses manure | 1 |
| Number of respondents | 78 |

**Table S.13.** Factors that prevent farmers from using co-compost on their crops

| Statement | Percentage (%) | |  |
| --- | --- | --- | --- |
|  | Yes | No | Valid number |
| I would need to do some more research before I can consider using co-compost in my farming systems | 62.3 | 37.7 | 77 |
| I would eat food fertilised with human excreta if the fertilizer was treated and certified | 49.4 | 50.6 | 77 |
| I would eat food fertilised with human excreta if the food was processed | 38.2 | 61.8 | 77 |
| I would eat food fertilised with human excreta if the food is going to be consumed cooked or boiled | 31.2 | 68.8 |  |
| Crops can die if fertilised with co-compost | 23.4 | 76.6 | 77 |
| The taste of crops and vegetables will change if I use co-compost | 57.1 | 42.9 | 77 |
| I use chemical fertilizer, so I don’t need co-compost | 80.5 | 19.5 | 77 |
| There are health risks associated with co-compost, so I will not use it | 57.1 | 42.9 | 77 |
| The smell of co-compost is a hindrance/disgusting | 94.7 | 5.3 | 77 |
| People will mock me or make fun of me | 68.4 | 31.6 | 77 |
| Taboo or religious belief | 56.0 | 44.0 | 77 |

**Table S.14.** Any other reasons for not using co-compost

| Reason | Number of respondents |
| --- | --- |
| Damages the soil | 1 |
| Dehumanising | 2 |
| Disgusting | 14 |
| Health risk | 3 |
| Not aware | 5 |
| Use cow manure | 2 |
| Total | 27 |

**Table S.15.** NEP scale Cronbach's Alpha factoring test for internal consistency

| Cronbach's Alpha | Cronbach's Alpha Based on Standardized Items | Number of N items |
| --- | --- | --- |
| 0.762 | 0.763 | 15 |

**Table S.16.** NEP scale exploratory factor analysis

| Item scale | Mean | Std. Dev | Cronbach's Alpha if Item Deleted |
| --- | --- | --- | --- |
| We are approaching the limit of the number of people the Earth can support | 3.57 | 1.01 | 0.74 |
| Humans have the right to modify the natural environment to suit their needs | 2.32 | 1.00 | 0.76 |
| When humans interfere with nature it often produces disastrous consequences | 3.65 | 0.90 | 0.74 |
| Human intelligence will ensure that we do not make Earth un–liveable | 2.49 | 1.02 | 0.75 |
| Humans are seriously abusing the environment | 3.87 | 1.04 | 0.74 |
| The Earth has plenty of natural resources if we just learn how to develop them | 2.18 | 0.85 | 0.77 |
| Plants and animals have as much right as humans to exist | 3.63 | 1.14 | 0.76 |
| The balance of nature is strong enough to cope with the impacts of modern industrial nations | 2.86 | 0.96 | 0.74 |
| Despite our special abilities, humans are still subject to the laws of nature | 3.91 | 0.85 | 0.76 |
| The so–called "ecological crisis" facing humankind has been greatly exaggerated | 3.08 | 1.09 | 0.76 |
| The Earth is like a spaceship with very limited room and resources | 3.32 | 1.04 | 0.74 |
| Humans were meant to rule over the rest of nature | 2.14 | 1.04 | 0.76 |
| The balance of nature is very delicate and easily upset | 3.57 | 0.82 | 0.74 |
| Humans will eventually learn enough about how nature works to be able to control it | 2.37 | 0.88 | 0.76 |
| If things continue on their present course, we will soon experience a major ecological catastrophe | 3.77 | 0.89 | 0.74 |
| Mean NEP rating | 3.12 | 0.47 |  |

**Table S.17.** The dimensionality of the NEP scale against hypothesized facets

| Factor | Initial eigenvalues (unrotated) | | | Extraction sums of squared loadings (varimax rotation) | | |
| --- | --- | --- | --- | --- | --- | --- |
|  | Total | % of variance | Cumulative % | Total | % of Variance | Cumulative % |
| 1 | 3.59 | 25.27 | 25.27 | 2.951 | 20.80 | 20.80 |
| 2 | 1.94 | 13.68 | 38.95 | 1.907 | 13.44 | 34.24 |
| 3 | 1.19 | 8.39 | 47.34 | 1.526 | 10.75 | 44.98 |
| 4 | 1.12 | 7.87 | 55.21 | 1.451 | 10.22 | 55.21 |
| 5 | 0.89 | 6.30 | 61.51 |  |  |  |
| 6 | 0.84 | 5.89 | 67.40 |  |  |  |
| 7 | 0.79 | 5.57 | 72.97 |  |  |  |
| 8 | 0.64 | 4.49 | 77.46 |  |  |  |
| 9 | 0.60 | 4.26 | 81.72 |  |  |  |
| 10 | 0.53 | 3.72 | 85.44 |  |  |  |
| 11 | 0.52 | 3.64 | 89.08 |  |  |  |
| 12 | 0.50 | 3.49 | 92.56 |  |  |  |
| 13 | 0.42 | 2.93 | 95.50 |  |  |  |
| 14 | 0.33 | 2.29 | 97.79 |  |  |  |
| 15 | 0.31 | 2.21 | 100.00 |  |  |  |

**Table S.18.** NEP scale factor loadings

| Variables/questions | 5 hypothesized facets | Component loadings | | | |
| --- | --- | --- | --- | --- | --- |
|  |  | 1 | 2 | 3 | 4 |
| 1 | Limits | **0.68** | -0.30 | 0.18 | 0.05 |
| 2 | Antianthro | **0.68** | -0.29 | 0.26 | 0.21 |
| 3 | Balance | **0.64** | -.026 | 0.18 | 0.23 |
| 4 | Antiexempt | **0.64** | -0.10 | 0.07 | 0.05 |
| 5 | Eco-crisis | **0.63** | -0.21 | 0.04 | 0.11 |
| 6 | Limits | **0.62** | 0.02 | -0.07 | -0.26 |
| 7 | Antianthro | **0.54** | 0.25 | -0.06 | 0.27 |
| 8 | Balance | **0.42** | **0.65** | 0.06 | 0.03 |
| 9 | Antiexempt | 0.22 | **0.58** | 0.11 | -0.06 |
| 10 | Eco-crisis | 0.09 | **0.56** | 0.04 | -0.07 |
| 11 | Limits | **0.38** | **-0.53** | 0.14 | -0.12 |
| 12 | Antianthro | **0.37** | **0.39** | **-0.35** | 0.30 |
| 13 | Balance | **0.47** | -0.19 | **-0.64** | **-0.51** |
| 14 | Antiexempt | **0.30** | 0.26 | **-0.37** | **0.35** |
| 15 | Eco-crisis | **0.36** | **0.41** | **0.45** | **-0.52** |

**Table S.19.** Exploratory factor analysis of the attitudinal questions or scale items

| Item scale/attitudinal question | Mean | Std. Dev | Cronbach's Alpha if Item Deleted |
| --- | --- | --- | --- |
| Do you think you have enough skills/knowledge/resources to use human excreta in your farming systems | 1.09 | 0.29 | 0.82 |
| Do you think human excreta can be treated so as to NOT pose a health risk? | 1.83 | 0.37 | 0.82 |
| Do you think treated human excreta contains pathogens (microorganisms that can cause diseases) when applied to crops? | 1.42 | 0.49 | 0.83 |
| Do you think pharmaceuticals/medicines can be found in crops growth with human excreta derived fertilizer like co-compost? | 1.37 | 0.48 | 0.85 |
| Do you think co-compost can be used to fertilise crops? | 1.77 | 0.42 | 0.80 |
| Do you think human urine can be used to fertilise crops? | 1.48 | 0.50 | 0.81 |
| Do you think human excreta should be disposed and never be reused | 1.51 | 0.50 | 0.81 |
| Would you buy fertilizer made from human excreta or co-compost? | 1.73 | 0.45 | 0.79 |
| Would you buy food that was fertilised with human urine? | 1.57 | 0.49 | 0.80 |
| Would you buy food that was fertilised with human faecal matter? | 1.66 | 0.47 | 0.79 |
| Do you think other people in general, would use human excreta in their fields to fertilize crops | 1.63 | 0.48 | 0.80 |
| Do you think other people in the market will buy food produced using co-compost as fertiliser? | 1.71 | 0.46 | 0.80 |
| Do you think your family members would eat food that was fertilised with human excreta? | 1.42 | 0.49 | 0.80 |
| Do you think your, neighbours, friends, relatives or other people would eat food that was fertilised with human excreta? | 1.59 | 0.49 | 0.79 |

**Table S.20.** Principal component analysis with varimax rotation to compute attitudinal scores

| Factor |  | Initial eigenvalues (unrotated) | | | Extraction sums of squared loadings (varimax rotation) | | |
| --- | --- | --- | --- | --- | --- | --- | --- |
|  |  | Total | % of variance | Cumulative % | Total | % of Variance | Cumulative % |
| 1 |  | 1.15 | 38.82 | 38.82 | 0.77 | 26.02 | 26.02 |
| 2 |  | 0.34 | 11.27 | 50.08 | 0.68 | 22.70 | 48.72 |
| 3 |  | 0.28 | 9.36 | 59.45 | 0.32 | 10.72 | 59.45 |
| 4 |  | 0.21 | 7.11 | 66.56 |  |  |  |
| 5 |  | 0.18 | 5.99 | 72.54 |  |  |  |
| 6 |  | 0.16 | 5.23 | 77.77 |  |  |  |
| 7 |  | 0.13 | 4.22 | 81.99 |  |  |  |
| 8 |  | 0.11 | 3.77 | 85.77 |  |  |  |
| 9 |  | 0.10 | 3.50 | 89.27 |  |  |  |
| 10 |  | 0.08 | 2.53 | 91.79 |  |  |  |
| 11 |  | 0.07 | 2.49 | 94.29 |  |  |  |
| 12 |  | 0.07 | 2.17 | 96.65 |  |  |  |
| 13 |  | 0.06 | 1.88 | 98.33 |  |  |  |
| 14 |  | 0.05 | 1.69 | 100.00 |  |  |  |

**Table S.21.** The named dimensionality of the attitudinal scale

| Item scale | Component 1 (high loadings on perceived behavioral control, production, market, subjective norms,) | Component 2 (high loadings on production. market attitudes and subjective norms) | Component 3 (low loadings of all questions) | |
| --- | --- | --- | --- | --- |
| Do you think you have enough skills/knowledge/resources to use human excreta in your farming systems | 0.81 |  | |  |
| Do you think human excreta can be treated so as to NOT pose a health risk? | 0.80 |  | |  |
| Do you think treated human excreta contains pathogens (microorganisms that can cause diseases) when applied to crops? | 0.77 |  | | 0.32 |
| Do you think pharmaceuticals/medicines can be found in crops growth with human excreta derived fertilizer like co-compost? | 0.74 |  | |  |
| Do you think co-compost can be used to fertilise crops? | 0.73 |  | | 0.31 |
| Do you think human urine can be used to fertilise crops? | 0.69 |  | |  |
| Do you think human excreta should be disposed and never be reused | 0.66 | -0.41 | | 0.43 |
| Would you buy fertilizer made from human excreta or co-compost? | 0.63 |  | |  |
| Would you buy food that was fertilised with human urine? | 0.59 |  | | -0.42 |
| Would you buy food that was fertilised with human faecal matter? | 0.54 | 0.31 | |  |
| Do you think other people in general, would use human excreta in their fields to fertilize crops | 0.41 |  | |  |
| Do you think other people in the market will buy food produced using co-compost as fertiliser? |  | 0.78 | | 0.32 |
| Do you think your family members would eat food that was fertilised with human excreta? |  |  | |  |
| Do you think your, neighbours, friends, relatives or other people would eat food that was fertilised with human excreta? | -0.32 | 0.50 | | 0.55 |


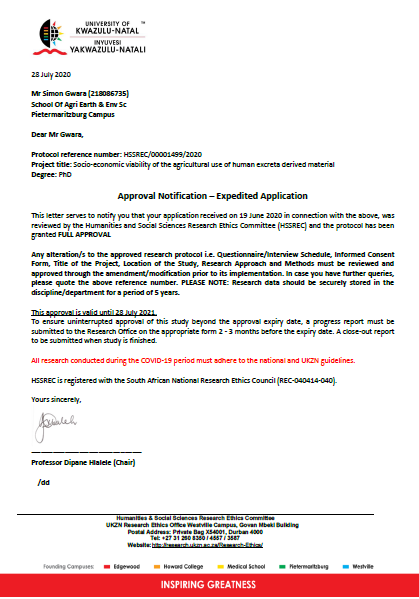


**Figure S.1.** Sampling Humanities and Social Sciences Research Ethics Committee (HSSREC) Ethical Clearance
